# Associations between interleukin-1 gene polymorphisms and sepsis risk: a meta-analysis

**DOI:** 10.1186/1471-2350-15-8

**Published:** 2014-01-16

**Authors:** An-qiang Zhang, Wei Pan, Jun-wei Gao, Cai-li Yue, Ling Zeng, Wei Gu, Jian-xin Jiang

**Affiliations:** 1 State Key Laboratory of Trauma, Burns and Combined Injury, Institute of Surgery Research, Daping Hospital, Third Military Medical University, Chongqing 400042, China

**Keywords:** Sepsis, *IL-1*, Polymorphism, Meta-analysis

## Abstract

**Background:**

Previous epidemiological studies have presented conflicting evidence regarding associations between interleukin-1 (*IL-1*) polymorphisms and sepsis susceptibility. We have performed a meta-analysis to evaluate possible associations between *IL-1* polymorphisms and sepsis risk.

**Methods:**

Eligible literature was retrieved from PubMed, Embase and Web of Knowledge databases until Jun 15, 2013. The pooled odds ratio (OR) and 95% confidence interval (CI) were calculated using random-effects model in the overall and subgroup analysis based on ethnicity, sepsis severity and quality score.

**Results:**

Eighteen studies addressing five *IL-1* polymorphisms were included in this meta-analysis. For *IL-1A-889* (rs1800587) polymorphism, significant association was observed in overall comparison for allelic effect (OR = 1.47, 95% CI = 1.01-2.13, P = 0.04). There were no significant associations between either *IL-1B-511* (rs16944) or *IL-1B-31* (rs1143627) and sepsis susceptibility in overall or subgroup analyses. For *IL-1B + 3594* (rs143634) polymorphism, genotype TT decreased sepsis risk in overall analysis (OR = 0.59, 95% CI = 0.36-0.97, P = 0.04), as well as in Caucasian (OR = 0.57, 95% CI = 0.34-0.95, P = 0.03) and sepsis (OR = 0.55, 95% CI = 0.31-0.97, P = 0.04) subgroup analysis. For *IL-1RN VNTR* polymorphism, significant association was observed in overall comparison for allelic effect (OR = 1.40, 95% CI = 1.01-1.95, P = 0.04). Furthermore, the effect sizes of *IL-1RN VNTR* on sepsis risk increased with disease severity (septic shock OR > severe sepsis OR > sepsis OR).

**Conclusions:**

Our meta-analysis indicated that *IL-1A-889, IL-1B + 3954* and *IL-1RN VNTR* might be associated with sepsis susceptibility. However, further studies with larger sample sizes and from homogenous populations would be necessary to validate these findings.

## Background

Sepsis is a complex clinical syndrome that results from a systemic inflammatory response to bacteria and/or bacterial products [1]. Though there have been many advances in the development of antibiotics and supportive care, sepsis remains a serious and deadly problem with high mortality rates worldwide [2]. Therefore, predictive markers to identify high-risk patients are urgently needed for early detection and preventive care of sepsis. Cytokines play vital roles in the regulation of host immune response, and altered expression of cytokines is proven to be involved the development of sepsis [3]. Previous research suggests that the variations in the genes encoding cytokines are also involved in the inflammatory responses and are responsible for inter-individual differences in susceptibility to sepsis and in its severity [4]. Delineating the variations in cytokine genes, and associated differences in response to sepsis might contribute to the development of new genetically tailored diagnostic and therapeutic interventions that may improve outcome in sepsis patients.

Interleukin-1 (*IL-1*) family is a critical mediator of immune response to sepsis with two agonists (IL-1α and IL-1β) and one antagonist (IL-1 receptor antagonist: IL-1ra) [5-7]. Excessive IL-1 production is directly linked to the development of shock, multi-organ system failure, and death in patients and animals with sepsis, systemic inflammatory response syndrome, and septic shock [7]. Clinical trials with recombinant IL-1ra in the treatment of sepsis showed the highest mortality rate in those patients treated with the largest dose of IL-1ra, suggesting that overproduction of either pro-inflammatory mediators (IL-1α and IL-1β) or anti-inflammatory cytokines (IL-1ra) might lead to organ dysfunction and even death [8].

*IL-1A*, *IL-1B* and *IL-1RN* genes (encoding IL-1α, IL-1β, and IL-1ra, respectively) are located next to each other within the cluster of human major histocompatibility complex in the q13-21 area of human chromosome 2 [9]. Five SNPs in *IL-1* genes have been most frequently studied in relation to sepsis risk: one SNP at promoter position −889 in the *IL-1A* gene, two SNPs at promoter position −511 and −31 and one SNP in exon 5 at position +3954 of the *IL-1B* gene and a variable number of tandem repeats (VNTR) of 86-bp sequence in intron 2 of *IL-1RN* gene, 5 alleles of which have been reported (1 to 5) corresponding to 4, 2, 5, 3 and 6 copies of 86-bp sequence, respectively. Thus, the *IL-1RN* alleles were further divided into two categories: long genotype (L: including alleles 1, 3, 4, and 5) and short genotype (2: allele 2 only). The genotypes were classified as L/L, L/2, and 2/2 [10].

Recently, a number of studies regarding the associations between *IL-1* polymorphisms and sepsis risk have been published [11-27]. However, the results reported from these studies are inconsistent and inconclusive. We performed a meta-analysis to further investigate the associations between *IL-1* polymorphisms and sepsis risk, which may help us to better clarify the effect of these polymorphisms on sepsis susceptibility.

## Methods

### Identification and eligibility of relevant study

Relevant articles were identified through a literature search using the keywords “*IL-1* or *interleukin-1* or *IL1*” and “sepsis or severe sepsis or septic shock” and “polymorphism or genetic variant or mutation” in Pubmed, Embase, and Web of Knowledge databases until Jun 15, 2013. All searched articles were retrieved and their references were checked for other relevant publications.

The inclusion criteria were: (a) studies evaluating the association between *IL-1* polymorphisms and sepsis risk, (b) case–control study or cohort design, (c) sufficient data (genotype distributions of cases and controls), and (d) studies written in English. We excluded reviews, comments and articles from which the necessary data could not be extracted nor obtained after contacting the authors. In cases of overlapping studies, only the study with the largest sample size was included.

To minimize the bias and improve the reliability, two researchers reviewed these articles with the inclusion and exclusion criteria independently and reached a consensus.

### Data extraction

Data extraction was independently performed by two investigators and discrepancies were settled by reaching a consensus. Information such as the first author’s name, publication year, country and ethnicity of participants, sepsis severity, genotyping method, genotype number or allele frequencies for cases and controls was collected from each study using a standardized data collection protocol. For studies including subjects of different populations, data were extracted separately.

### Qualitative assessment

Quality assessment was performed with the Newcastle-Ottawa quality assessment scale (NOS) [28]. A ‘star system’ has been used to judge data quality based on three broad perspectives: the selection, comparability and outcome of interest. Stars are added up to compare the study quality in a quantitative fashion. The scores ranged from 0 to 9 stars. Studies with scores of 7 stars or greater were considered to be of high quality. Based on these criteria, the content validity was evaluated by J-WG and C-LY, and any disagreement was resolved via discussions between and or with the other authors for adjudication.

### Statistical analysis

The associations between *IL-1* polymorphisms and sepsis risk were estimated by calculating pooled ORs and 95%CI under the dominant (BB + AB vs. AA), recessive (BB vs. AB + AA), and allelic (B vs. A) genetic models respectively (B represented minor allele, A represented major allele). A random-effects model, using Mantel-Haenszel method, was used to calculate pooled ORs. The significance of pooled ORs was determined with Z tests (P < 0.05 was considered statistically significant). Departure from Hardy-Weinberg equilibrium (HWE) in controls was tested via a Chi-square test at a significance level of P < 0.05. Heterogeneity between studies was assessed using the Cochrane Q test and the I^2^ statistic [29]. Multivariate meta-regression was performed to explore potential sources of heterogeneity among the following covariates: ethnicity (divided into Asian, Caucasian and others), sepsis severity (divided into sepsis, severe sepsis and septic shock), sources of controls (divided into community- or hospital-based) and sample size using REML method of random-effects when the number of studies was more than or equal to 10 [30]. Subgroup analyses were carried out based on ethnicity, severity of sepsis, and quality score. To assess the stability of the pooled results, sensitivity analysis was performed by sequentially excluding individual studies one by one. The publication bias was examined by funnel plot [31] and Egger’s test [32]. To adjust the values for multiple comparisons in subgroup analyses, we used the Benjamini-Hochberg (BH) step-up correction method, which control the false discovery rate (FDR) [33]. All statistical tests were performed using Review Manager 5.2 (The Cochrane Collaboration, Oxford, UK) and STATA11.0 software (StataCorp LP, College Station, Texas, USA).

## Result

### Characteristics of eligible studies

**Figure 1:**
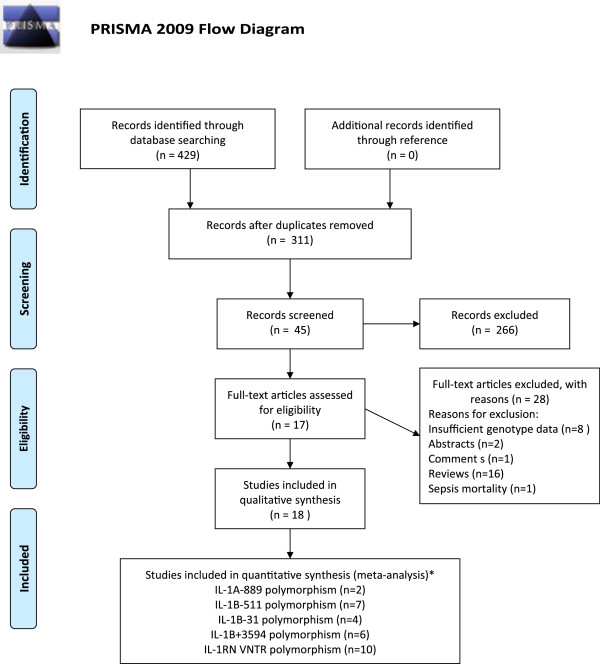
**Flow of study identification, inclusion, and exclusion.** *represents that certain studies will appear in more than one group when they are divided by polymorphism.

A total of 429 articles were identified update to Jun 15, 2013 (107 from PubMed, 144 from Embase, and 178 from Web of Knowledge). After scanning the abstracts and checking the full-text articles, seventeen articles met the inclusion criteria. Furthermore, two populations containing Caucasian and Black subjects were included in one article by Johnson et al. [13] which was therefore considered as two independent studies (referred to as Johnson-1 and Johnson-2) in the following data analysis. Thus, 18 studies addressing five polymorphisms in IL-1 gene were included in our meta-analysis (Flow diagram shown in Figure 1).

The characteristics of included studies are listed in Table 1. Of these 18 studies, eight were conducted in Caucasian, six in Asian, two in Hispanic, one in African-American, and one in Jewish/Arabic populations. Except for one study for *IL-1B + 3594* and another for *IL-1RN VNTR*, the genotype frequency distributions of others were in agreement with HWE (Table 1). Quality scores for individual studies ranged from 6 to 9, with 78% (14 of 18) of the studies being classified as high quality (score ≥ 7) (Additional file 1: Table S1). The classical polymerase chain reaction (PCR) and PCR-restriction fragment length polymorphism (PCR-RFLP) were the most commonly used genotyping method in these studies.

**Table 1:** Characteristics of studies included in the meta-analysis

**Author/Year**	**Country**	**Ethnicity**	**Sepsis severity**	**Sources of control**	**Genotyping method**	**Quality score**	**Case/control**	**Case**			**Control**			**P**_**HWE**_
								**AA**	**AB**	**BB**	**AA**	**AB**	**BB**	
**IL1α-889C/T**														
Davis 2010	America	Hispanic	Sepsis	Community	TaqMan	7	28/52	9	16	3	24	24	4	0.55
Gu 2010	China	Asian	Sepsis	Hospital (trauma)	Probes	8	165/141	114	42	9	107	31	3	0.67
**IL-1B-511G/A**														
Johnson-1 2012	America	Caucasian	Sepsis	Hospital (noninfected)	Mass-array	9	245/263	103	114	28	101	120	42	0.53
Johnson-2 2012	America	African-American	Sepsis	Hospital (noninfected)	Mass-array	9	93/88	28	36	29	26	43	19	0.88
Wan 2012	China	Asian	Sepsis	Hospital (transplanted)	PCR-RFLP	7	21/60	11	6	4	12	30	18	0.94
Shimada 2011	Japan	Asian	Sepsis	Hospital (critical ill)	Probes	7	123/101	33	60	30	35	46	20	0.49
Davis 2010	America	Hispanic	Sepsis	Community	TaqMan	7	28/53	17	6	5	19	29	5	0.20
Gu 2010	China	Asian	Sepsis	Hospital (trauma)	Probes	8	167/140	57	83	27	34	61	45	0.15
Watanabe 2005	Japan	Asian	Sepsis	Hospital (SIRS)	Probes	6	68/45	19		49^a^	20		25^a^	0.86
**IL-1B-31C/T**														
Shimada 2011	Japan	Asian	Sepsis	Hospital (critical ill)	Probes	8	122/101	36	58	28	34	47	20	0.61
Emonts 2010	Netherlands	Caucasian	Sepsis	Community	SNaPshot	7	84/456	10	37	37	57	214	185	0.69
Gu 2010	China	Asian	Sepsis	Hospital (trauma)	Probes	8	163/144	29	106	28	66	60	18	0.45
Barber 2004	America	Caucasian	SS	Hospital (burn)	Pyro	8	36/123	11	13	12	21	54	48	0.39
**IL-1B + 3594C/T**														
Johnson-1 2012	America	Caucasian	Sepsis	Hospital (noninfected)	Mass-array	9	245/263	148	89	8	158	88	17	0.32
Johnson-2 2012	America	African-American	Sepsis	Hospital (noninfected)	Mass-array	9	93/88	61	29	3	65	20	3	0.36
Zhang 2005	China	Asian	SSH	Hospital (ASP)	PCR-RFLP	6	33/76	27	6	0	68	8	0	0.63
Balding 2003	Ireland	Caucasian	Sepsis	Community	PCR-RFLP	6	183/389	113	65	5	240	125	24	0.16
Treszl 2003	Hungary	Caucasian	Sepsis	Hospital (VLBW)	PCR-RFLP	6	33/70	20	11	2	41	25	4	0.94
Fang 1999	Germany	Caucasian	SS	Community	PCR	7	93/112	55	31	7	68	33	11	0.03
**IL-1RN VNTR L/2**														
Zapata-Tarres 2013	Mexico	Hispanic	SSH	Hospital (ALL)	PCR	8	22/35	6	11	5	23	11	1	0.82
Wan 2012	China	Asian	Sepsis	Hospital (transplanted)	PCR-RFLP	7	21/60	17	4	0	51	9	0	0.53
Davis 2010	America	Hispanic	Sepsis	Community	TaqMan	7	28/50	12	14	2	27	20	3	0.78
Garcia-Segarra 2007	Spain	Caucasian	SS and SSH	Community	PCR	7	114/80	59	52	3	30	41	9	0.37
Watanabe 2005	Japan	Asian	Sepsis	Hospital (SIRS)	PCR	6	68/45	59		9^a^	39		6^a^	0.54
Bessler 2004	Israel	Jews/Arabs	Sepsis	Community	PCR	7	34/61	18	10	6	33	16	12	0.002
Balding 2003	Ireland	Caucasian	Sepsis	Community	PCR-RFLP	6	183/389	88	70	25	198	160	31	0.87
Arnalich 2002	Spain	Caucasian	SS	Hospital (CAP)	PCR	8	78/186	32	33	13	88	81	17	0.79
Ma 2002	China	Asian	Sepsis	Community	PCR	7	60/60	26	27	7	36	21	3	0.98
Fang 1999	Germany	Caucasian	SS	Community	PCR	7	93/261	37	43	13	152	92	17	0.54

SS: severe sepsis; SSH: septic shock; SIRS: systemic inflammatory response syndrome; ASP: acute severe pancreatitis; VLBW: very-low-birth-weight infants; ALL: acute lymphoblastic leukemia; CAP: community-acquired pneumonia; L signifies any long allele embracing allele 1, 3, 4, or 5; ^a^represents the number of AB + BB genotype (A represents the major allele, B represents the minor allele); P_HWE_ < 0.05 is as a significant deviation from HWE.

### Quantitative data synthesis

#### *IL-1A-889* (rs1800587) polymorphism

Only two studies [15,17] with high quality containing 193 cases and 193 controls evaluated the association of the *IL-1A-889* polymorphism (rs1800587) with sepsis risk. In overall comparison, a significant association was observed for allelic effect (OR = 1.47, 95% CI = 1.01-2.13, P = 0.04). There were no statistically significant associations between this polymorphism and sepsis risk under dominant or recessive models (Additional file 2: Figure S1, Table 2).

**Table 2:** Summary of meta-analysis results

	**Dominant model**					**Recessive model**					**Allelic model**				
	**n**	**OR (95% CI)**	**P-value**	**P**_**het**_	**I**^**2**^	**n**	**OR (95% CI)**	**P-value**	**P**_**het**_	**I**^**2**^	**n**	**OR (95% CI)**	**P-value**	**P**_**het**_	**I**^**2**^
**IL1A-889C/T**															
**Total (sepsis)**	2	1.49 (0.95-2.33)	0.08	0.65	0%	2	2.06 (0.75-5.67)	0.16	0.56	0%	**2**	**1.47 (1.01-2.13)**	**0.04**	**0.98**	**0%**
**Ethnicity**															
Asian	1	1.41 (0.85-2.34)	0.19			1	2.65 (0.70-10.0)	0.15			1	1.47 (0.94-2.30)	0.09		
Others	1	1.81 (0.69-4.74)	0.23			1	1.44 (0.30-6.94)	0.65			1	1.46 (0.74-2.87)	0.28		
**IL1B-511G/A**															
**Total (sepsis)**	7	0.81 (0.53-1.24)	0.33	<0.01	68%	6	0.89 (0.53-1.50)	0.66	<0.01	67%	6	0.82 (0.60-1.11)	0.20	<0.01	69%
**Ethnicity**															
Asian	4	0.85 (0.39-1.87)	0.69	<0.01	81%	3	0.67 (0.29-1.55)	0.35	0.02	73%	3	0.74 (0.38-1.34)	0.29	<0.01	84%
Caucasian	1	0.86 (0.60-1.23)	0.40			1	0.68 (0.41-1.13)	0.14			1	0.84 (0.65-1.08)	0.18		
Others	2	0.63 (0.24-1.66)	0.35	0.09	66%	2	1.73 (0.95-3.14)	0.07	0.76	0%	2	0.98 (0.58-1.65)	0.93	0.18	44%
**Quality score**															
≥7	6	0.72 (0.48-1.9)	0.12	0.02	64%	6	0.89 (0.53-1.50)	0.66	<0.01	67%	6	0.82 (0.60-1.11)	0.20	<0.01	69%
<7	1	2.06 (0.94-4.55)	0.07												
**IL1B-31C/T**															
**Total (high quality)**	4	1.29 (0.54-3.06)	0.57	<0.01	86%	4	1.16 (0.86-1.56)	0.34	0.69	0%	4	1.18 (0.78-1.77)	0.43	<0.01	78%
**Ethnicity**															
Asian	2	2.19 (0.70-6.90)	0.18	<0.01	89%	2	1.33 (0.84-2.09)	0.22	0.69	0%	2	1.53 (0.91-2.57)	0.11	0.04	77%
Caucasian	2	0.73 (0.33-1.61)	0.43	0.15	52%	2	1.04 (0.70-1.56)	0.85	0.40	0%	2	0.90 (0.57-1.43)	0.65	0.14	55%
**Sepsis severity**															
Sepsis	3	1.75 (0.74-4.11)	0.20	<0.01	84%	3	1.24 (0.89-1.72)	0.20	0.85	0%	3	1.37 (0.93-2.00)	0.11	0.03	72%
Severe sepsis	1	0.47 (0.20-1.10)	0.08			1	0.78 (0.36-1.71)	0.54			1	0.68 (0.40-1.15)	0.15		
Septic shock															
**IL1B + 3594C/T**															
**Total**	6	1.06 (0.86-1.31)	0.57	0.79	0%	**5**	**0.59 (0.36-0.97)**	**0.04**	**0.81**	**0%**	6	0.97 (0.81-1.15)	0.72	0.71	0%
**Ethnicity**															
Asian	1	1.89 (0.60-5.96)	0.28								1	1.80 (0.60-5.41)	0.30		
Caucasian	4	1.00 (0.80-1.25)	0.99	0.74	0%	**4**	**0.57 (0.34-0.95)**	**0.03**	**0.74**	**0%**	4	0.92 (0.76-1.11)	0.37	0.99	0%
Others	1	1.48 (0.78-2.81)	0.23			1	0.94 (0.19-4.81)	0.95			1	1.34 (0.77-2.33)	0.31		
**Sepsis severity**															
Sepsis	4	1.04 (0.83-1.30)	0.75	0.71	0%	**4**	**0.55 (0.31-0.97)**	**0.04**	**0.73**	**0%**	4	0.95 (0.78-1.15)	0.59	0.64	0%
Severe sepsis	1	1.07 (0.61-1.87)	0.82			1	0.75 (0.28-2.01)	0.56			1	0.98 (0.62-1.54)	0.93		
Septic shock	1	1.89 (0.60-5.96)	0.28								1	1.80 (0.60-5.41)	0.30		
**Quality score**															
≥7	3	1.08 (0.82-1.42)	0.57	0.55	0%	3	0.63 (0.34-1.14)	0.13	0.71	0%	3	0.98 (0.78-1.23)	0.89	0.47	0%
<7	3	1.04 (0.75-1.43)	0.83	0.56	0%	3	0.53 (0.23-1.25)	0.15	0.37	0%	3	0.95 (0.72-1.24)	0.70	0.49	0%
**IL1RN VNTR L/2**															
**Total**	9	1.39 (0.97-1.98)	0.07	0.01	59%	8	1.67 (0.93-3.02)	0.09	0.04	54%	**8**	**1.40 (1.01-1.95)**	**0.04**	**<0.01**	**72%**
**Ethnicity**															
Asian	3	1.55 (0.89-2.68)	0.12	0.58	0%	1	2.51 (0.62-10.21)	0.20			2	1.69 (1.01-2.84)	0.05	0.64	0%
Caucasian	4	1.16 (0.72-1.88)	0.54	<0.01	75%	4	1.39 (0.66-2.92)	0.38	0.02	71%	4	1.17 (0.77-1.78)	0.45	<0.01	82%
Others	2	2.68 (0.84-8.50)	0.09	0.12	58%	2	3.17 (0.40-25.32)	0.28	0.15	52%	2	2.27 (0.78-6.58)	0.13	0.05	73%
**Sepsis severity**															
Sepsis	5	1.26 (0.95-1.67)	0.11	0.69	0%	**4**	**1.85 (1.12-3.04)**	**0.02**	**0.82**	**0%**	**4**	**1.31 (1.05-1.64)**	**0.02**	**0.70**	**0%**
Severe sepsis	**2**	**1.67 (1.03-2.71)**	**0.04**	**0.18**	**44%**	**2**	**2.16 (1.25-3.72)**	**0.01**	**0.77**	**0%**	**2**	**1.60 (1.18-2.17)**	**<0.01**	**0.25**	**24%**
Septic shock	**1**	**5.11 (1.59-16.46)**	**0.01**			**1**	**10.00 (1.08-92.49)**	**0.04**			**1**	**4.00 (1.72-9.31)**	**<0.01**		
**Quality score**															
≥7	7	1.53 (0.95-2.47)	0.08	<0.01	67%	7	1.62 (0.72-3.65)	0.24	0.02	61%	7	1.46 (0.96-2.21)	0.08	<0.01	75%
<7	2	1.11 (0.79-1.55)	0.55	0.84	0%	1	1.83 (1.04-3.20)	0.03			1	1.22 (0.93-1.60)	0.14		

Random effects model is used whether or not heterogeneity exists; others contain African-American, Hispanic, and Jews/Arabs; n: the number of studies involved. OR: odds ratio; CI: confidence interval; P-value: P value of pooled OR; P_het_: P of heterogeneity; Statistically significant results (P < 0.05) are highlighted in bold.

#### *IL-1B-511* (rs16944) and −*31* (rs1143627) polymorphisms

Seven studies [12-15,17,34] totaling 745 cases and 750 controls were identified in order to investigate the association between *IL-1B-511* polymorphism (rs16944) and sepsis risk. Overall, the results showed no associations under any genetic model. Removal of the low score study ([34] with score = 6), did not alter these results. In addition, there was no association in any subgroup analysis, either based on ethnicity or sepsis severity under any genetic model (Additional file 2: Figure S2, Table 2).

For the *IL-1B-31* polymorphism (rs1143627), four studies [14-16,22] with high quality totaling 405 cases and 824 controls were identified. Similar to *IL-1B-511* polymorphism, there were no significant associations detected between rs1143627 and sepsis risk overall or in any subgroup analysis for any genetic model (Additional file 2: Figure S3, Table 2).

#### *IL-1B + 3594* (rs143634) polymorphism

**Figure 2:**
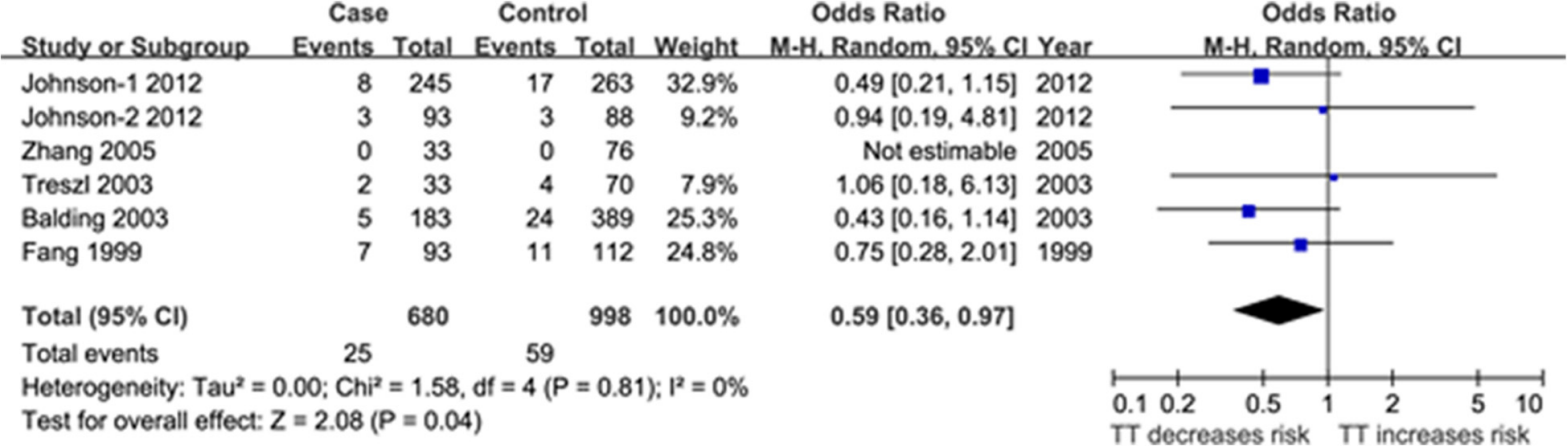
**Forest plot of sepsis susceptibility associated with *IL-1B + 3594 *polymorphism under the recessive model (TT vs. CT + CC).** The squares and horizontal line represent the individual study-specific OR and 95% CI. Area of squares is proportional to the weight of the individual study to the overall pooled OR. The diamond at the bottom of the graph represents the pooled OR and 95% CI. Events^1^: Number of individuals with TT genotype. OR, odds ratio; CI, confidence interval.

Six studies [13,20,23,24,27] containing 680 cases and 998 controls were identified that evaluated the association between the *IL-1B + 3594* polymorphism (rs143634) and sepsis risk. In the overall comparison, the *IL-1B + 3594* polymorphism was significantly associated with sepsis risk in the recessive effect (OR = 0.59, 95% CI = 0.36-0.97, P = 0.04, P_FDR_ = 0.12) (Figure 2, Additional file 2: Figure S4). In the subgroup analyses based on ethnicity and sepsis subtype, the significant results persisted in the Caucasian populations (OR = 0.57, 95% CI = 0.34-0.95, P = 0.03, P_FDR_ = 0.09) and in sepsis subgroup patients (OR = 0.55, 95% CI = 0.31-0.97, P = 0.04, P_FDR_ = 0.12). However, the negative results for Asians, severe sepsis, and septic shock group patients might be not reliable because only one study was performed in each subgroup (Table 2). After the exclusion of the study by Fang et al. [27], whose genotypic distribution in controls deviated from HWE (P_HWE_ = 0.03), the results did not vary significantly (OR = 0.55, 95% CI = 0.31-0.97, P = 0.04, P_FDR_ = 0.12 for overall studies; OR = 0.51, 95% CI = 0.28-0.93, P = 0.03, P_FDR_ = 0.09 for Caucasian populations). Furthermore, the results of stratified analysis based on quality score were not statistically significant.

#### *IL-1RN VNTR* polymorphism

**Figure 3:**
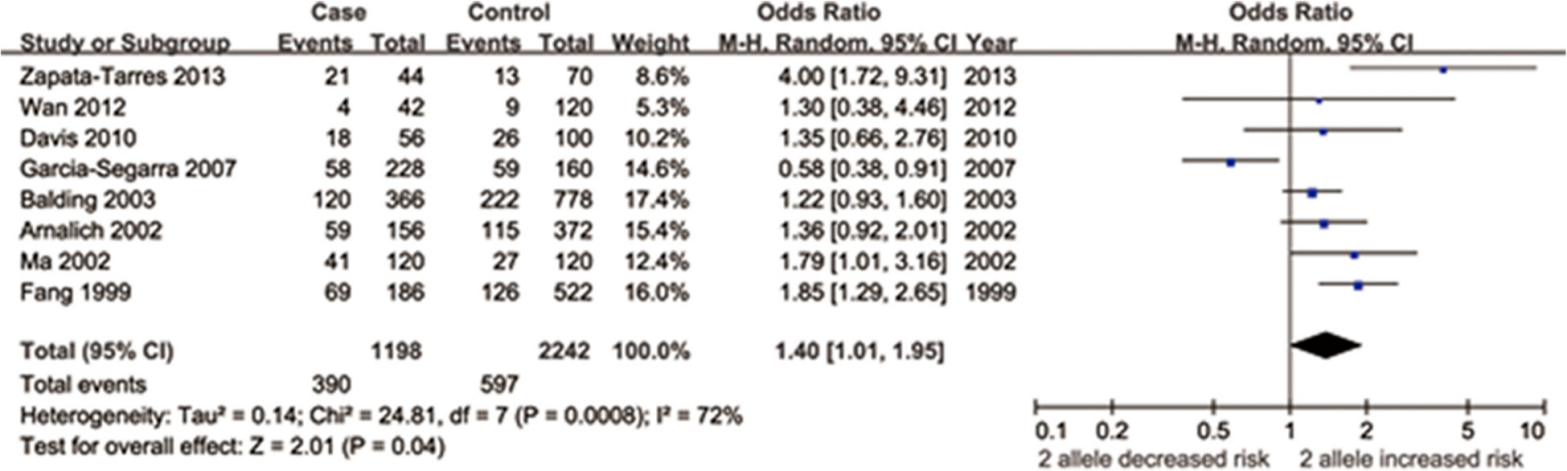
**Forest plot of sepsis susceptibility associated with *IL-1RN VNTR *polymorphism under the allelic model (2 vs. L).** The squares and horizontal line represent the individual study-specific OR and 95% CI. Area of squares is proportional to the weight of the individual study to the overall pooled OR. The diamond at the bottom of the graph represents the pooled OR and 95% CI. Events^1^: Number of the 2 allele. OR, odds ratio; CI, confidence interval.

For the *IL-1RN VNTR* polymorphism, ten studies [11,12,17,19,21,24-27,34] comprised of 701 cases and 1227 controls were identified. However, one study by Bessler et al. [21] that deviated from HWE (P_HWE_ = 0.002) was not included into the final meta-analysis. The overall results suggested there was statistically significant association of this polymorphism with sepsis risk under allelic model (OR = 1.40, 95% CI = 1.01-1.95, P = 0.04, P_FDR_ = 0.12) (Figure 3, Additional 1: Additional file 2: Figure S5). A similar trend was observed in Asian patients (OR = 1.69, 95% CI = 1.01-2.84) although the results were not statistically significant (P = 0.05, P_FDR_ = 0.15). Further stratified analyses based on sepsis severity showed that the effect sizes of *IL-1RN VNTR* on sepsis risk increased with disease severity (septic shock patients > severe sepsis patients > sepsis patients). These findings needed to be interpreted with caution since only one or two studies were included under the three genetic models. Furthermore, stratified analysis based on quality score indicated that there was not significant association in the high quality studies (Quality score ≥7) (Table 2).

### Heterogeneity analysis

As shown in Table 2, obvious evidence (P < 0.1 and I^2^ > 50%) for heterogeneity between studies was found for *IL-1B-511* or *IL-1RN VNTR* under all three genetic models, and for *IL-1B-31* under dominant and allelic models. No evidence for heterogeneity between studies was found for *IL-1A-889* or *IL-1B + 3594* under any genetic model.

To explore potential sources of between-study heterogeneity in our assessment of *IL-1RN VNTR* polymorphism and sepsis susceptibility, meta-regression was conducted for all three genetic models. The confounding factors included ethnicity, sepsis severity, sources of controls, and sample size. However, the result did not indicate that any of these potential factors was a major source of heterogeneity (P-value for regression all > 0.05) (Additional file 3: Table S2-S4). Indeed, the heterogeneity might be attributable to other factors, which regrettably remain undefined due to insufficient data. For the other two polymorphisms (*IL-1B-511* and −*31*), meta-regression was not performed due to the small number of included studies.

### Sensitivity analysis

We removed one study per time from the overall pooled analysis to evaluate the influence of the removed data set on the pooled ORs. The corresponding pooled ORs under any genetic model were not materially altered for *IL-1A-889*, *IL-1B-511*, and −*31*, respectively (Additional file 4: Table S5-S7). However, for *IL-1B + 3594*, two studies (Johnson-1 et al. [13] and Balding et al. [24]) altered the corresponding statistical P value of association under the recessive model (Additional file 4: Table S8b). For *IL-1RN VNTR*, one study (Garcia-Segarra et al. [19]) was identified as the main cause of heterogeneity. After exclusion of this study, the heterogeneity no longer existed, moreover, positive association was increased under all three genetic models (Additional file 4: Table S9).

### Publication bias

**Figure 4:**
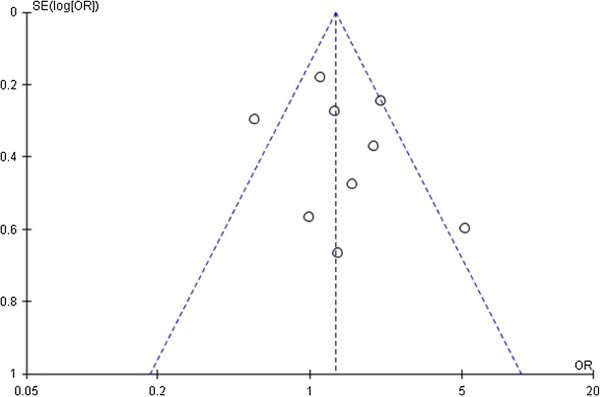
**Funnel plot of sepsis susceptibility associated with *IL-1RN VNTR *under the dominant model (2/2 + L/2 vs. L/L).** Each circle represents an independent study; for each study the OR was plotted against the standard error of the log of the OR. Center dotted line represents the pooled OR and sloping dotted lines represent the 95% CI of the pooled OR. OR, odds ratio; SE, standard error; CI, confidence interval.

**Figure 5:**
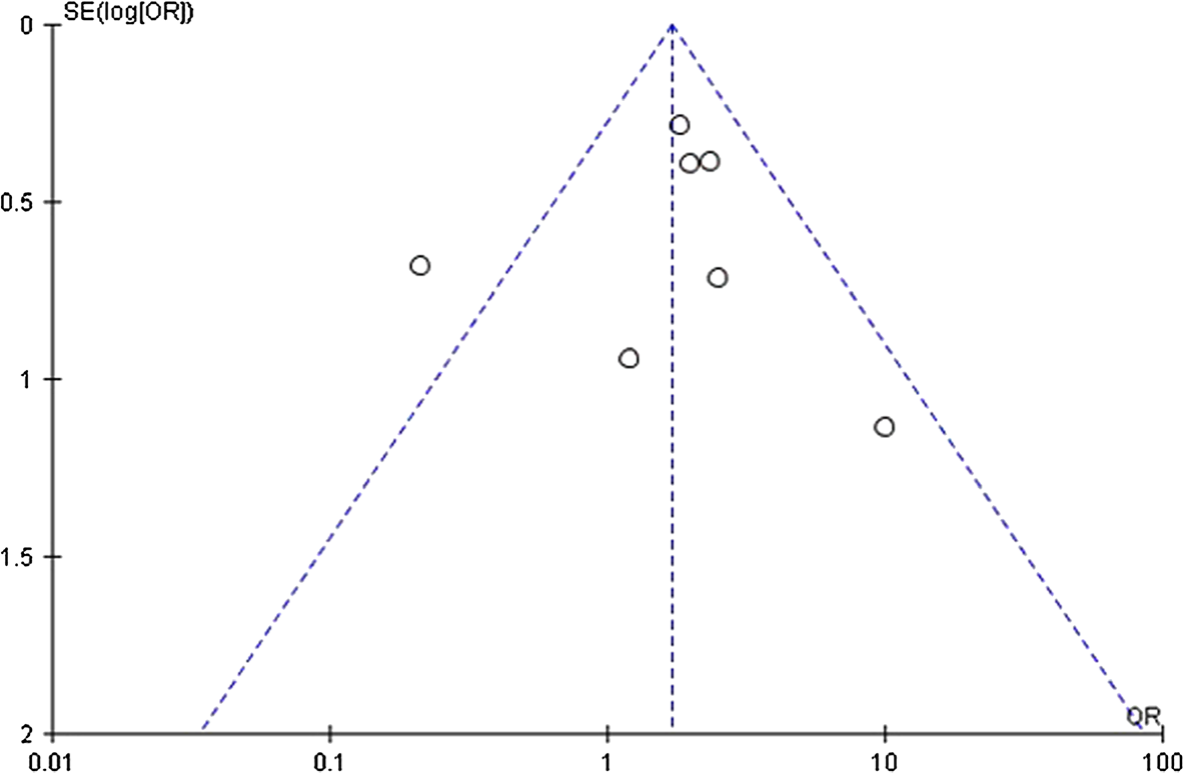
**Funnel plot of sepsis susceptibility associated with *IL-1RN VNTR *under the recessive model (2/2 vs. L/2 + L/L).** Each circle represents an independent study; for each study the OR was plotted against the standard error of the log of the OR. Center dotted line represents the pooled OR and sloping dotted lines represent the 95% CI of the pooled OR. OR, odds ratio; SE, standard error; CI, confidence interval.

**Figure 6:**
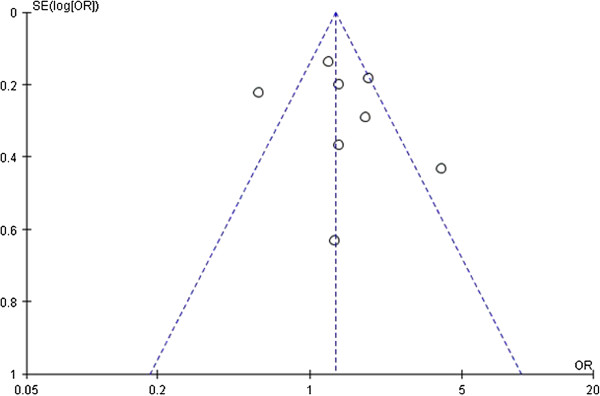
**Funnel plot of sepsis susceptibility associated with *IL-1RN VNTR *under the allelic model (2 vs. L).** Each circle represents an independent study; for each study the OR was plotted against the standard error of the log of the OR. Center dotted line represents the pooled OR and sloping dotted lines represent the 95% CI of the pooled OR. OR, odds ratio; SE, standard error; CI, confidence interval.

Publication bias was examined by funnel plots and Egger’s test under all genetic models. After combining all studies, a little asymmetry was observed for *IL-1RN VNTR* polymorphism (Figures 4, 5 and 6), but Egger’s test did not show evidence of publication bias (dominant: P = 0.519, recessive: P = 0.724, allelic: P = 0.640). For *IL-1A-889*, *IL-1B-511*, *-31*, and *+3594* polymorphisms, publication bias testing was not performed due to the small number of included studies (n < 9).

## Discussion

In this meta-analysis, two polymorphisms (*IL-1B + 3594* and *IL-1RN VNTR*) were significantly associated with sepsis susceptibility in overall comparison and subgroup analyses based on sepsis severity, whereas *IL-1A-889* polymorphism influenced sepsis risk only in overall comparison. In contrast, no association was observed between either *IL-1B-511* or −*31* and sepsis risk in overall comparison or subgroup analyses.

*IL-1B + 3594* polymorphism is a coding synonymous variant located in exon 5 of *IL-1B*. The transition from C to T does not change amino acid coding but may lead to an inactivation of the original splicing donor site. The alternative splicing results in a premature stop codon or exon skipping and produces a truncated protein that is likely to be rapidly degraded or functionally inactive [35]. The *IL1B + 3954 T* allele has been reported to increase the production of IL-1β protein in response to LPS-stimulation [36]. The results of our meta-analysis revealed that individuals with variant genotype (TT) were less susceptible to sepsis than individuals with CC or CT genotypes in overall comparison and Caucasian population, which was inconsistent with higher IL-1β levels associated with increased risk of sepsis. Considering that sepsis is a multifactorial trait and the impact of the inflammatory cytokine on sepsis progress may be modulated by other environmental and genetic factors, more studies should be conducted to clarify the role of *IL-1B + 3594* polymorphism in the etiology of sepsis.

For the *IL-1RN VNTR* polymorphism, functional analysis in vitro has shown that the *IL-1RN*-2 allele correlated with high IL-1ra level and an even more elevated IL-1β level. This resulted in the lowest IL-1ra/IL-1β ratio and was associated with a strengthened and prolonged inflammatory response [37,38]. In addition, Arnalich et al. [26] found that *IL-1RN*-2 allele causes a decreased production of serum IL-1Ra in patients with severe sepsis and ethnically matched healthy controls compared with 1 allele. These findings implied that the 86 bp VNTR polymorphism of the *IL-1RN* has potential roles in regulating the immune response and contributed to the pathogenesis of inflammatory diseases. Similarly, our meta-analysis indicated a significant association with risk of sepsis in overall comparison and stratified analysis based on different sepsis severity, which was not observed in ethnicity subgroup analysis. This indicates that the discrepancies between the overall and ethnicity subgroup analyses may be attributed to the diverse genetic backgrounds and environmental factors influencing sepsis risk in different ethnic populations. Further studies with larger samples and from homogeneous populations are warranted to further evaluate the role of the *IL-1RN VNTR* polymorphism in sepsis risk in different populations.

*IL-1A-889* polymorphism, a C-to-T point mutation in its 5’ regulatory region, affected over-expression of IL-1α [35]. In the current meta-analysis, there was significant association with sepsis susceptibility in overall comparison. However, only two studies were included into this meta-analysis with small samples. Thus, this finding needed to be confirmed with larger samples.

The −*511* and −*31* polymorphisms, which are located at position 511 bp and 31 bp upstream of the transcriptional start site, have been shown to influence the transcriptional activity and expression of the *IL-1B* gene. The presence of variant allele of *IL-1B-511* markedly increased endotoxin-induced production of IL-1β [36]. Moreover, *IL1B-31C/T* substitution located in the TATA box motif has been found to markedly affect the binding of several transcription factors and thereby affect the transcriptional activity [39]. In addition, two bi-allelic SNPs (−*511*, and −*31*) within the human *IL-1B* promoter region have been reported to affect LPS-induced IL-1β transcription in vitro and IL-1β plasma levels in healthy adults [40]. Although IL-1β plays an important role in sepsis and IL-1β is frequently over-expressed in sepsis, our meta-analysis indicated no significant association between the *IL-1B-511* and −*31* polymorphisms and sepsis risk, suggesting that IL-1β expression might influence sepsis progression via mechanisms other than regulation by the two promoter polymorphisms. Several other factors, such as *IL-1RN* and *NLRP3* may also regulate IL-1β expression [41,42]. Further studies are needed to test these hypotheses.

Sepsis is a multifactorial trait and the impact of the inflammatory cytokine on sepsis progress may be modulated by, age, gender and some other environmental factors [43]. Interpretation of the pooled results has been hampered by the fact that several different clinical conditions such as sepsis, severe sepsis, or septic shock have been analyzed together according to the published consensus definitions for sepsis [44]. In our stratified analyses, we also examined that whether the effect of polymorphisms differed depending on the way in which sepsis was described (sepsis, severe sepsis, or septic shock). Our results indicated the positive association was observed for *IL-1B + 3594C/T* in sepsis group patients, for *IL-1RN VNTR* polymorphism in sepsis, severe sepsis, and septic shock group patients, respectively. These findings indicated that the way in which sepsis was described affected the association between *IL-1* polymorphisms and susceptibility to sepsis. However, what also needs to be pointed out is that these significant associations were derived from only one to two studies and thus the result should be interpreted with caution because of the relatively small sample size.

Heterogeneity is an important problem when interpreting the results of our meta-analysis. In this study, significant heterogeneity was found for the *IL-1B-511*, *-31*, and *IL-1RN VNTR* polymorphisms. Meta-regression did not seem to reveal the potential sources of heterogeneity with the introduction of covariates containing ethnicity, sepsis severity, sources of controls, sample size, which indicated that the heterogeneity might be attributable to other factors, which regrettably remain undefined due to insufficient data. Moreover, we carried out sensitivity analysis. Removal of each study did not seem to alter the relationships with sepsis risk and heterogeneity for *IL-1B-511* and −*31*, suggesting the reliability of these results. However, removing one study by Garcia-Segarra et al. for *IL-1RN VNTR* obviously decreased the heterogeneity and increased positive association under all genetic models, which indicated this study may be the main cause of heterogeneity.

Another important problem is publication bias. Because meta-analyses review quantitative data from numerous studies, the publication bias effect of the literature incorporated in the study can bias the mea-analytic outcome. For *IL-1RN VNTR* polymorphism, although the Egger’s test did not show significant publication bias for sepsis risk, we found the shape of the funnel plot was slightly asymmetrical. Thus, the results should be interpreted cautiously and more studies are still needed to confirm the findings from this meta-analysis.

Some limitations of this meta-analysis should be pointed out. First, the number and sample size of the included studies was limited, some unpublished reports, non-English articles and studies without sufficient information were not included in our meta-analysis, which might bias the pooled results. Second, although the results in stratified analysis were more meaningful, there were a small number of studies in each stratum, thus limiting the interpretation of these analyses. Third, as none of the studies included in this meta-analysis considered the effect of gene-gene/environment involved in the pathogenesis of sepsis, this issue could not be addressed. Fourth, the overall outcome was based on unadjusted data, where as a more precise analysis stratified by variables such as age, sex, type of infection etc. could not be performed due to limitations of the data which also restricted our ability to detect possible sources of heterogeneity. Fifth, stratified analyses based on the different sources of controls were not performed due to small number of studies in each subgroup; the inclusion of studies with varied control populations might increase the probability of typeIerror and bias the pooled results. Finally, as we only focused on the associations between *IL-1* polymorphisms and sepsis susceptibility in the present study, the significance was limited. To better illuminate the role of IL-1 polymorphisms in outcome of sepsis, it would also be important to perform meta-analyses on the associations between polymorphisms and severity or sepsis-related mortality in future studies.

## Conclusions

To our knowledge, this is the first study that quantitatively synthesized the association between the *IL-1* polymorphisms and sepsis. The results demonstrated that the *IL-1A-889C/T*, *IL-1B-3594C/T*, and *IL-1RN VNTR* polymorphisms had significant associations with the risk of sepsis, although some results were limited by the small number of studies. However, no significant association existed between *IL-1B-511*, *-31* and sepsis risk. Further studies with large samples and homogeneous population are needed to evaluate their associations with sepsis risk.

## Abbreviations

IL-1: Interleukin-1; IL-1RN: Interleukin-1 receptor antagonist; VNTR: Variable number of tandem repeats; OR: Odds ratio; CI: Confidence interval; HWE: Hardy-Weinberg equilibrium; NOS: Newcastle-Ottawa quality assessment scale; PCR-RFLP: Polymerase chain reaction-restriction fragment length polymorphism.

## Competing interests

The authors declare that they have no competing interests.

## Authors’ contributions

A-QZ and WP was the main researcher for this study and took part in conceptualization, literature review, data extraction, analysis, writing of the manuscript. J-WG, C-LY and LZ were involved in software used, data analysis and final editing. WG did to guide the statistical analysis and manuscript editing. J-XJ planned the study, wrote the protocol and was involved in the genetic and clinical aspects of data analyses and revised the manuscript. All authors read and approved the final manuscript.

## Pre-publication history

The pre-publication history for this paper can be accessed here:

http://www.biomedcentral.com/1471-2350/15/8/prepub

## Supplementary Material

Additional file 1: Table S1 Assessment of study quality. Click here for file

Additional file 2: Figure S1-S5 Forest plot of sepsis susceptibility associated with *IL-1* polymorphisms under a random-effect model. Click here for file

Additional file 3: Table S2-S4 Meta-regression of sepsis susceptibility associated with *IL-1RN VNTR* polymorphism under a random-effect model with covariates of ethnicity, sepsis severity, sources of controls and sample size. Click here for file

Additional file 4: Table S5-S9 Sensitivity analysis result for IL-1 polymorphisms. Click here for file
