# Supplementary material for: Associations between interleukin-1 gene polymorphisms and sepsis risk: a meta-analysis
Source: BMC Med Genet. 2014 Jan 16;15:8. doi: 10.1186/1471-2350-15-8 (PMC3901334; doi:10.1186/1471-2350-15-8)
Supplement: Additional file 2: Figure S1-S5 — Forest plot of sepsis susceptibility associated with IL-1 polymorphisms under a random-effect model. [file 1471-2350-15-8-S2.pdf]

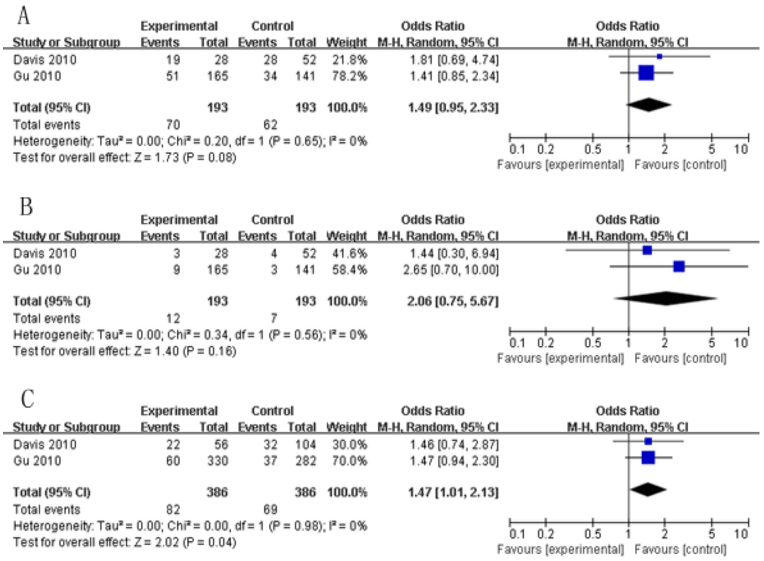

Figure S1. Forest plot of sepsis susceptibility associated with *IL-1A-889* polymorphism under a random-effect model. A: CT+TT vs. CC, B: TT vs. CT+CC, C: T vs. C. The squares and horizontal line represent the individual study-specific OR and 95%CI. Area of squares is proportional to the weight of the individual study to the overall pooled OR. The diamond at the bottom of the graph represents the pooled OR and 95%CI. OR, odds ratio; CI, confidence interval.

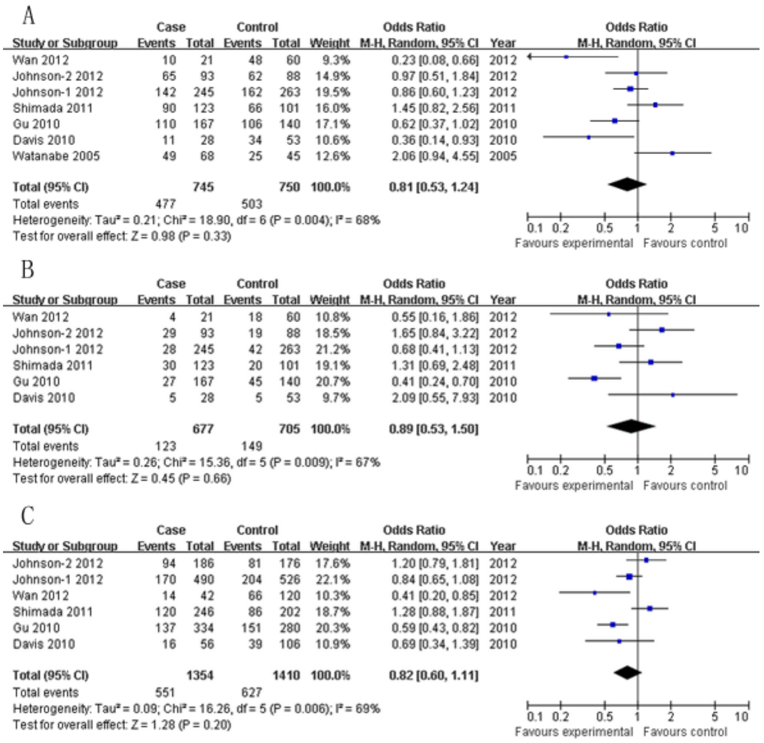

Figure S2. Forest plot of sepsis susceptibility associated with *IL-1B-511* polymorphism under a random-effect model. A: GA+GG vs. AA, B: GG vs. GA+AA, C: G vs. A. The squares and horizontal line represent the individual study-specific OR and 95%CI. Area of squares is proportional to the weight of the individual study to the overall pooled OR. The

diamond at the bottom of the graph represents the pooled OR and 95%CI. OR, odds ratio; CI, confidence interval.

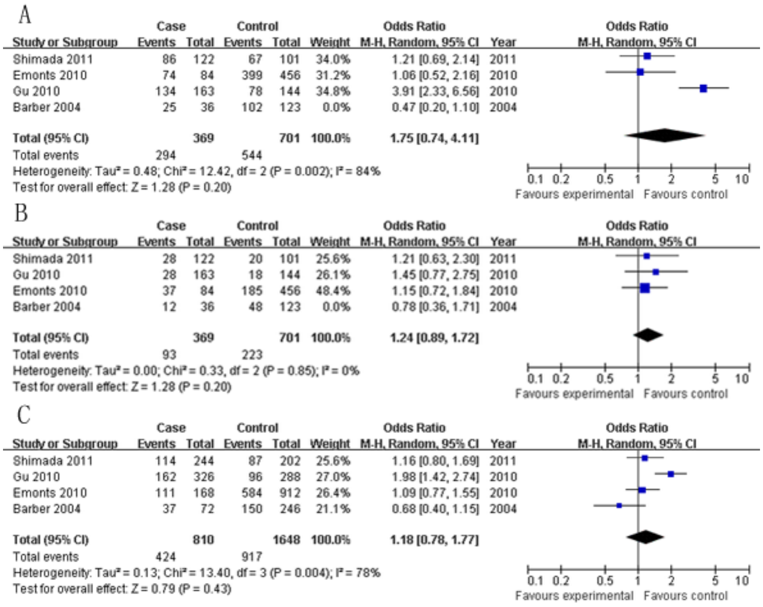

Figure S3. Forest plot of sepsis susceptibility associated with *IL-1B-31* polymorphism under a random-effect model. A: CT+TT vs. CC, B: TT vs. CT+CC, C: T vs. C. The squares and horizontal line represent the individual study-specific OR and 95%CI. Area of squares is proportional to the weight of the individual study to the overall pooled OR. The diamond at the bottom of the graph represents the pooled OR and 95%CI. OR, odds ratio; CI, confidence interval.

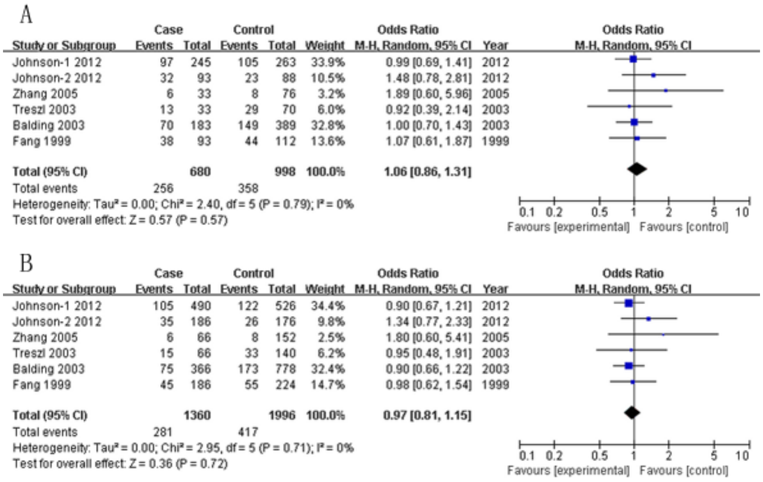

Figure S4. Forest plot of sepsis susceptibility associated with *IL-1B+3594* polymorphism under a random-effect model. A: CT+TT vs. CC, B: T vs. C. The squares and horizontal line represent the individual study-specific OR and 95%CI. Area of squares is proportional to the weight of the individual study to the overall pooled OR. The diamond at the bottom of the graph represents the pooled OR and 95%CI. OR, odds ratio; CI, confidence interval.

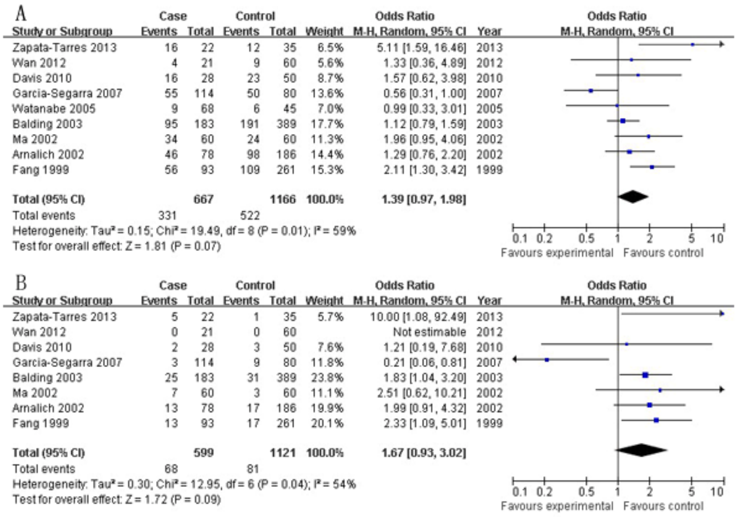

Figure S5. Forest plot of sepsis susceptibility associated with *IL-1RN VNTR* polymorphism under a random-effect model. A: L/2+2/2 vs. L/L, B: 2/2 vs. L/2+L/L. The squares and horizontal line represent the individual study-specific OR and 95%CI. Area of squares is proportional to the weight of the individual study to the overall pooled OR. The diamond at the bottom of the graph represents the pooled OR and 95%CI. OR, odds ratio; CI, confidence interval.
