# Supplementary material for: Associations between interleukin-1 gene polymorphisms and sepsis risk: a meta-analysis
Source: BMC Med Genet. 2014 Jan 16;15:8. doi: 10.1186/1471-2350-15-8 (PMC3901334; doi:10.1186/1471-2350-15-8)
Supplement: Additional file 3: Table S2-S4 — Meta-regression of sepsis susceptibility associated with IL-1RN VNTR polymorphism under a random-effect model with covariates of ethnicity, sepsis severity, sources of controls and sample size. [file 1471-2350-15-8-S3.docx]

**Table S2. Meta-regression of sepsis susceptibility associated with *IL-1RN VNTR* polymorphism under a random-effect model with covariates of ethnicity, sepsis severity, sources of controls and sample size (L/2+2/2 vs. L/L).**

Number of obs =9

REML estimate of between-study variance tau2 =0.09

% residual variation due to heterogeneity I^2^ =72.43%

Proportion of between-study variance explained Adj R^2^ =-81.03%

Joint test for all covariates Model F (4, 3) =0.37

With knapp-Hartung modification Prob > F =0.82

| **logor** | **Coefficient** | **Standard error** | **t** | **P-value** |
| --- | --- | --- | --- | --- |
| **Ethnicity** | 0.15(-0.40~0.70) | 0.20 | 0.74 | 0.50 |
| **Severity of sepsis** | -0.11(-0.46~0.24) | 0.13 | -0.87 | 0.43 |
| **Source of controls** | 0.14(-0.62~0.89) | 0.27 | 0.50 | 0.64 |
| **Sample size** | -0.0003(-0.002~0.002) | 0.001 | -0.44 | 0.68 |
| **Constant** | -0.002(-1.75~1.75) | 0.63 | -0.00 | 0.99 |

**Table S3. Meta-regression of sepsis susceptibility associated with *IL-1RN VNTR* polymorphism under a random-effect model with covariates of ethnicity, sepsis severity, sources of controls and sample size (2/2 vs. L/2+L/L).**

Number of obs =7

REML estimate of between-study variance tau2 =0.78

% residual variation due to heterogeneity I^2^ =72.58%

Proportion of between-study variance explained Adj R^2^ =-246.95%

Joint test for all covariates Model F (4, 3) =0.54

With knapp-Hartung modification Prob > F =0.73

| **logor** | **Coefficient** | **Standard error** | **t** | **P-value** |
| --- | --- | --- | --- | --- |
| **Ethnicity** | 0.13(-3.32~3.58) | 0.80 | 0.16 | 0.89 |
| **Severity of sepsis** | -0.52(-2.54~1.49) | 0.47 | -1.12 | 0.38 |
| **Source of controls** | 1.06(-3.39~5.51) | 1.03 | 1.02 | 0.41 |
| **Sample size** | -0.0001(-0.01~0.01) | 0.003 | -0.04 | 0.97 |
| **Constant** | -0.12(-9.87~9.63) | 2.27 | -0.05 | 0.96 |

**Table S4. Meta-regression of sepsis susceptibility associated with *IL-1RN VNTR* polymorphism under a random-effect model with covariates of ethnicity, sepsis severity, sources of controls and sample size (2 vs. L).**

Number of obs =8

REML estimate of between-study variance tau2 =0.15

% residual variation due to heterogeneity I^2^ =81.41%

Proportion of between-study variance explained Adj R^2^ =-72.92%

Joint test for all covariates Model F (4, 3) =0.43

With knapp-Hartung modification Prob > F =0.79

| **logor** | **Coefficient** | **Standard error** | **t** | **P-value** |
| --- | --- | --- | --- | --- |
| **Ethnicity** | 0.14(-0.69~0.98) | 0.26 | 0.55 | 0.62 |
| **Severity of sepsis** | -0.15(-0.67~0.37) | 0.16 | -0.90 | 0.43 |
| **Source of controls** | -0.30(-0.85~1.45) | 0.36 | 0.82 | 0.47 |
| **Sample size** | -0.0003(-0.003~0.003) | 0.001 | -0.27 | 0.81 |
| **Constant** | -0.08(-2.65~2.49) | 0.81 | -0.10 | 0.93 |
