# Supplementary material for: Associations between interleukin-1 gene polymorphisms and sepsis risk: a meta-analysis
Source: BMC Med Genet. 2014 Jan 16;15:8. doi: 10.1186/1471-2350-15-8 (PMC3901334; doi:10.1186/1471-2350-15-8)
Supplement: Additional file 4: Table S5-S9 — Sensitivity analysis result for IL-1 polymorphisms. [file 1471-2350-15-8-S4.docx]

**Table S5a. Sensitivity analysis result for IL-1A/-889C/T (CT+TT vs. CC).**

| **Study** | **OR** | **95% CI** | | ***p* value** |
| --- | --- | --- | --- | --- |
|  |  | **Lower limit** | **Upper limit** |  |
| Davis 2010 | 1.41 | 0.85 | 2.34 | 0.19 |
| Gu 2010 | 1.81 | 0.69 | 4.74 | 0.23 |

**Table S5b. Sensitivity analysis result for IL-1A/-889C/T (TT vs. CT+CC).**

| **Study** | **OR** | **95% CI** | | ***p* value** |
| --- | --- | --- | --- | --- |
|  |  | **Lower limit** | **Upper limit** |  |
| Davis 2010 | 2.65 | 0.70 | 10.00 | 0.15 |
| Gu 2010 | 1.44 | 0.30 | 6.94 | 0.65 |

**Table S5c. Sensitivity analysis result for IL-1A/-889C/T (T vs. C).**

| **Study** | **OR** | **95% CI** | | ***p* value** |
| --- | --- | --- | --- | --- |
|  |  | **Lower limit** | **Upper limit** |  |
| Davis 2010 | 1.47 | 0.94 | 2.30 | 0.09 |
| Gu 2010 | 1.46 | 0.74 | 2.87 | 0.28 |

**Table S6a. Sensitivity analysis result for IL-1B/-511G/A (GA+AA vs. GG).**

| **Study** | **OR** | **95% CI** | | ***p* value** |
| --- | --- | --- | --- | --- |
|  |  | **Lower limit** | **Upper limit** |  |
| Johnson-1 2012 | 0.78 | 0.44 | 1.37 | 0.39 |
| Johnson-2 2012 | 0.78 | 0.47 | 1.28 | 0.32 |
| Wan 2012 | 0.92 | 0.63 | 1.35 | 0.68 |
| Shimada 2011 | 0.73 | 0.46 | 1.15 | 0.17 |
| Davis 2010 | 0.89 | 0.58 | 1.37 | 0.61 |
| Gu 2010 | 0.85 | 0.51 | 1.39 | 0.51 |
| Watanabe 2005 | 0.72 | 0.48 | 1.09 | 0.12 |

**Table S6b. Sensitivity analysis result for IL-1B/-511G/A (AA vs. GA+GG).**

| **Study** | **OR** | **95% CI** | | ***p* value** |
| --- | --- | --- | --- | --- |
|  |  | **Lower limit** | **Upper limit** |  |
| Johnson-1 2012 | 0.97 | 0.49 | 1.91 | 0.92 |
| Johnson-2 2012 | 0.76 | 0.45 | 1.30 | 0.32 |
| Wan 2012 | 0.95 | 0.53 | 1.69 | 0.86 |
| Shimada 2011 | 0.81 | 0.45 | 1.49 | 0.50 |
| Davis 2010 | 0.81 | 0.47 | 1.39 | 0.45 |
| Gu 2010 | 1.08 | 0.68 | 1.70 | 0.76 |

**Table S6c. Sensitivity analysis result for IL-1B/-511G/A (A vs. G).**

| **Study** | **OR** | **95% CI** | | ***p* value** |
| --- | --- | --- | --- | --- |
|  |  | **Lower limit** | **Upper limit** |  |
| Johnson-1 2012 | 0.80 | 0.53 | 1.22 | 0.30 |
| Johnson-2 2012 | 0.76 | 0.54 | 1.06 | 0.10 |
| Wan 2012 | 0.89 | 0.66 | 1.20 | 0.43 |
| Shimada 2011 | 0.74 | 0.55 | 1.01 | 0.06 |
| Davis 2010 | 0.84 | 0.60 | 1.17 | 0.30 |
| Gu 2010 | 0.90 | 0.65 | 1.23 | 0.50 |

**Table S7a. Sensitivity analysis result for IL-1B/-31C/T (CT+TT vs. CC).**

| **Study** | **OR** | **95% CI** | | ***p* value** |
| --- | --- | --- | --- | --- |
|  |  | **Lower limit** | **Upper limit** |  |
| Shimada 2011 | 1.29 | 0.36 | 4.56 | 0.69 |
| Emonts 2010 | 1.36 | 0.43 | 4.28 | 0.60 |
| Gu 2010 | 0.90 | 0.53 | 1.54 | 0.71 |
| Barber 2004 | 1.75 | 0.74 | 4.11 | 0.20 |

**Table S7b. Sensitivity analysis result for IL-1B/-31C/T (TT vs. CT+CC).**

| **Study** | **OR** | **95% CI** | | ***p* value** |
| --- | --- | --- | --- | --- |
|  |  | **Lower limit** | **Upper limit** |  |
| Shimada 2011 | 1.14 | 0.81 | 1.61 | 0.44 |
| Emonts 2010 | 1.16 | 0.78 | 1.72 | 0.46 |
| Gu 2010 | 1.08 | 0.77 | 1.53 | 0.64 |
| Barber 2004 | 1.24 | 0.89 | 1.72 | 0.20 |

**Table S7c. Sensitivity analysis result for IL-1B/-31C/T (T vs. C).**

| **Study** | **OR** | **95% CI** | | ***p* value** |
| --- | --- | --- | --- | --- |
|  |  | **Lower limit** | **Upper limit** |  |
| Shimada 2011 | 1.17 | 0.66 | 2.08 | 0.60 |
| Emonts 2010 | 1.19 | 0.67 | 2.12 | 0.55 |
| Gu 2010 | 1.00 | 0.76 | 1.33 | 0.98 |
| Barber 2004 | 1.37 | 0.93 | 2.00 | 0.11 |

**Table S8a. Sensitivity analysis result for IL-1B/+3594C/T (CT+TT vs. CC).**

| **Study** | **OR** | **95% CI** | | ***p* value** |
| --- | --- | --- | --- | --- |
|  |  | **Lower limit** | **Upper limit** |  |
| Johnson-1 2012 | 1.10 | 0.86 | 1.42 | 0.45 |
| Johnson-2 2012 | 1.02 | 0.82 | 1.27 | 0.85 |
| Zhang 2005 | 1.04 | 0.84 | 1.29 | 0.70 |
| Balding 2003 | 1.10 | 0.85 | 1.41 | 0.48 |
| Treszl 2003 | 1.07 | 0.87 | 1.33 | 0.52 |
| Fang 1999 | 1.06 | 0.85 | 1.33 | 0.60 |

**Table S8b. Sensitivity analysis result for IL-1B/+3594C/T (TT vs. CT+CC).**

| **Study** | **OR** | **95% CI** | | ***p* value** |
| --- | --- | --- | --- | --- |
|  |  | **Lower limit** | **Upper limit** |  |
| Johnson-1 2012 | 0.65 | 0.36 | 1.19 | 0.16 |
| Johnson-2 2012 | 0.57 | 0.34 | 0.95 | 0.03 |
| Zhang 2005 | 0.59 | 0.36 | 0.97 | 0.04 |
| Balding 2003 | 0.66 | 0.37 | 1.17 | 0.16 |
| Treszl 2003 | 0.56 | 0.34 | 0.94 | 0.03 |
| Fang 1999 | 0.55 | 0.31 | 0.97 | 0.04 |

**Table S8c. Sensitivity analysis result for IL-1B/+3594C/T (T vs. C).**

| **Study** | **OR** | **95% CI** | | ***p* value** |
| --- | --- | --- | --- | --- |
|  |  | **Lower limit** | **Upper limit** |  |
| Johnson-1 2012 | 1.01 | 0.81 | 1.25 | 0.96 |
| Johnson-2 2012 | 0.94 | 0.78 | 1.12 | 0.48 |
| Zhang 2005 | 0.95 | 0.80 | 1.14 | 0.60 |
| Balding 2003 | 1.00 | 0.81 | 1.24 | 0.98 |
| Treszl 2003 | 0.97 | 0.81 | 1.16 | 0.74 |
| Fang 1999 | 0.97 | 0.80 | 1.17 | 0.73 |

**Table S9a. Sensitivity analysis result for IL-1RN VNTR (L/2+2/2 vs. L/L).**

| **Study** | **OR** | **95% CI** | | ***p* value** |
| --- | --- | --- | --- | --- |
|  |  | **Lower limit** | **Upper limit** |  |
| Zapata-Tarres 2013 | 1.27 | 0.92 | 1.75 | 0.15 |
| Wan 2012 | 1.40 | 0.96 | 2.04 | 0.08 |
| Davis 2010 | 1.38 | 0.93 | 2.04 | 0.11 |
| Garcia-Segarra 2007 | 1.55 | 1.16 | 2.08 | <0.01 |
| Watanabe 2005 | 1.43 | 0.98 | 2.10 | 0.07 |
| Balding 2003 | 1.47 | 0.95 | 2.28 | 0.09 |
| Arnalich 2002 | 1.42 | 0.93 | 2.16 | 0.10 |
| Ma 2002 | 1.33 | 0.90 | 1.97 | 0.15 |
| Fang 1999 | 1.28 | 0.88 | 1.86 | 0.20 |

**Table S9b. Sensitivity analysis result for IL-1RN VNTR (2/2 vs. L/2+L/L).**

| **Study** | **OR** | **95% CI** | | ***p* value** |
| --- | --- | --- | --- | --- |
|  |  | **Lower limit** | **Upper limit** |  |
| Zapata-Tarres 2013 | 1.52 | 0.85 | 2.71 | 0.16 |
| Wan 2012 | 1.67 | 0.93 | 3.02 | 0.09 |
| Davis 2010 | 1.72 | 0.90 | 3.28 | 0.10 |
| Garcia-Segarra 2007 | 2.07 | 1.44 | 2.98 | <0.01 |
| Balding 2003 | 1.62 | 0.72 | 3.65 | 0.24 |
| Arnalich 2002 | 1.60 | 0.75 | 3.39 | 0.22 |
| Ma 2002 | 1.58 | 0.81 | 3.08 | 0.18 |
| Fang 1999 | 1.54 | 0.73 | 3.22 | 0.25 |

**Table S9c. Sensitivity analysis result for IL-1RN VNTR (2 vs. L).**

| **Study** | **OR** | **95% CI** | | ***p* value** |
| --- | --- | --- | --- | --- |
|  |  | **Lower limit** | **Upper limit** |  |
| Zapata-Tarres 2013 | 1.27 | 0.93 | 1.72 | 0.13 |
| Wan 2012 | 1.41 | 1.00 | 2.00 | 0.05 |
| Davis 2010 | 1.41 | 0.98 | 2.04 | 0.06 |
| Garcia-Segarra 2007 | 1.57 | 1.23 | 1.99 | <0.01 |
| Balding 2003 | 1.46 | 0.96 | 2.24 | 0.08 |
| Arnalich 2002 | 1.42 | 0.96 | 2.12 | 0.08 |
| Ma 2002 | 1.36 | 0.94 | 1.96 | 0.10 |
| Fang 1999 | 1.33 | 0.92 | 1.93 | 0.13 |
